# Lipopolysaccharides and Cellular Senescence: Involvement in Atherosclerosis

**DOI:** 10.3390/ijms231911148

**Published:** 2022-09-22

**Authors:** Kaori Suzuki, Etsuo A. Susaki, Isao Nagaoka

**Affiliations:** 1 Department of Host Defense and Biochemical Research, Juntendo University Graduate School of Medicine, Bunkyo-ku 113-8421, Tokyo, Japan; 2 Department of Biochemistry and Systems Biomedicine, Juntendo University Graduate School of Medicine, Bunkyo-ku 113-8421, Tokyo, Japan; 3 Faculty of Medical Science, Juntendo University, 6-8-1 Hinode, Urayasu-Shi 279-0013, Chiba, Japan

**Keywords:** cellular senescence, senescence-associated secretory phenotype (SASP), lipopolysaccharide (LPS), endothelial cells, atherosclerosis

## Abstract

Atherosclerosis is a chronic inflammatory disease of the vascular walls related to aging. Thus far, the roles of cellular senescence and bacterial infection in the pathogenesis of atherosclerosis have been speculated to be independent of each other. Some types of macrophages, vascular endothelial cells, and vascular smooth muscle cells are in a senescent state at the sites of atherosclerotic lesions. Likewise, bacterial infections and accumulations of lipopolysaccharide (LPS), an outer-membrane component of Gram-negative bacteria, have also been observed in the atherosclerotic lesions of patients. This review introduces the integration of these two potential pathways in atherosclerosis. Previous studies have suggested that LPS directly induces cellular senescence in cultured monocytes/macrophages and vascular cells. In addition, LPS enhances the inflammatory properties (senescence-associated secretory phenotype [SASP]) of senescent endothelial cells. Thus, LPS derived from Gram-negative bacteria could exaggerate the pathogenesis of atherosclerosis by inducing and enhancing cellular senescence and the SASP-associated inflammatory properties of specific vascular cells in atherosclerotic lesions. This proposed mechanism can provide novel approaches to preventing and treating this common age-related disease.

## 1. Introduction

Atherosclerosis is a chronic inflammatory disease of the vascular walls involving multiple cell types during its pathogenesis and progression [1]. In the early stage, vascular endothelial cells (ECs) are activated by oxidized low-density lipoprotein (LDL) in serum and upregulate the expression of chemokines, such as monocyte chemoattractant protein-1 (MCP-1) and adhesion molecules (intercellular adhesion molecule-1 [ICAM-1] and vascular cellular adhesion molecule-1 [VCAM-1]). On the other hand, ECs exposed to oxidized LDL downregulate the expression and activity of endothelial nitric oxide synthase (eNOS), leading to decreased NO production and vasorelaxation. Monocytes are then recruited to the vascular surface by EC-derived chemokines and bind with ECs via the interaction of the integrins of monocytes and the ICAM-1/VCAM-1 of ECs [2]. The EC-bound monocytes enter the subendothelial space (intima) and differentiate into proinflammatory macrophages. In the progression stage, the monocyte-derived macrophages in plaque release chemokines (CCL2, CCL5, and CXCL1) and proteases (matrix metalloproteinases [MMPs] and cathepsins), thereby promoting the migration of vascular smooth muscle cells (VSMCs) from media to intima, where both the monocyte-derived macrophages and VSMCs transform into foam cells that accelerate plaque progression and instability. Finally, the foam cells undergo necrosis in advanced plaques, and VSMCs lose their proliferation activity, leading to rupture and thrombosis.

In addition to the well-documented cellular processes described, the involvement of cellular senescence and bacterial infection has been speculated in atherosclerosis. The accumulation of both senescent cells and bacterial components has been identified in human atherosclerotic lesions [3,4,5,6]. However, their contributions and mechanistic links in the pathogenesis of atherosclerosis have not been fully elucidated. Here, we introduce recent studies, including our own, to discuss the role of cellular senescence and bacterial infection in atherosclerosis, particularly focusing on the senescence-inducing and enhancing effects of lipopolysaccharide (LPS), an outer-membrane component of Gram-negative bacteria, on vascular cells and macrophages.

## 2. Atherosclerosis and Cellular Senescence

Cellular senescence was initially defined by Hayflick et al. as the irreversible growth arrest of human somatic cells after long-term culture [7], which was revealed later to be associated with the telomere shortening that occurs at each cell division [8]. In addition to the process of cell growth arrest, senescent cells have numerous characteristic features, such as an enlarged and flattened morphology, increased β-galactosidase activity, and the increased expression of cell cycle arrest-related molecules, such as cyclin-dependent kinase inhibitors (p16/INK4 and p21/WAF-1) and cell cycle checkpoint protein p53 [9]. Although β-galactosidase, a lysosomal enzyme, has an optimum acidic pH (pH 4.0–4.5) in normal cellular conditions, long-term-cultured human fibroblasts were found to demonstrate specific enzyme activity at pH 6.0, referred to as senescence-associated β-galactosidase (SA-β-Gal) activity [10]. This increased SA-β-Gal activity is widely accepted as a hallmark of senescent cells in culture and tissue specimens [11].

Cellular senescence was initially found to be induced intrinsically in long-term cultures. However, it has since been revealed that senescence is also induced as a cellular response to DNA damage, triggered by the extrinsic stimulation of cells with oxidizing agents, oncogenic signaling, or tumorigenic factors, such as transforming growth factor-β (TGF-β) and tumor necrosis factor-α (TNF-α) [12,13,14]. This has led to an understanding that cellular senescence is an antitumor mechanism to inhibit the proliferation of DNA-damaged cells generated by intrinsic and extrinsic stimulation [15]. In contrast, senescent cells were also found to secrete various inflammatory factors, a state referred to as senescence-associated secretory phenotype (SASP) [15,16]. SASP is characterized by the upregulated expression of proinflammatory molecules, including cytokines (IL-6 and IL-1α/β as representatives), chemokines (MCP-1 and IL-8), growth factors (insulin-like growth factor and vascular endothelial growth factor), adhesion molecules (ICAM-1 and VCAM-1), extracellular cell matrix components (fibronectin), and proteases (MMPs and elastases) [15]. The expression of these SASP molecules is mainly regulated by the NF-κB and MAPK pathways in many types of senescent cells [17,18,19,20]. The NF-κB p65 subunit accumulates on the chromatin of senescent cells and p65 knockdown compromises senescence [19]. Notably, the concept that senescent cells contribute to the pathogenesis of age-associated inflammatory diseases is now widely accepted since senescent cells are increased in patients exhibiting many age-associated diseases, while the inflammatory molecules produced by senescent cells closely overlap with those involved in the pathogenesis of age-associated inflammatory disorders [21,22].

The pathogenesis of atherosclerosis involves characteristic changes in ECs, VSMCs, monocytes/macrophages, and other immune cells. The involvement of senescent ECs in atherosclerosis was hypothesized early in 1984 [23]; the author speculated that cholesterol and hypertension could damage the endothelium [24], while undamaged ECs may be stimulated to start proliferating and thus undergo replicative senescence [23]. Later, the SASP of ECs was speculated to play a role in atherosclerosis since senescent ECs were shown to produce many cytokines (IL-1, IL-6, IL-8, IL-15, MCP-1, and TNF-α), adhesion molecules (ICAM-1 and VCAM-1), plasminogen activator inhibitor-1 (PAI-1), growth factors (vascular endothelial growth factor and TGF-β), and proteases (MMPs) in vitro. Minamino et al. reported that senescent ECs (SA-β-gal^+^ factor VIII^+^ cells) were observed in atherosclerotic lesions of the coronary arteries but not in the internal mammary arteries of patients with ischemic heart disease [3]. They also showed that in senescent human aortic endothelial cells that were generated by introducing a dominant-negative form of telomeric repeat-binding factor 2 (TRF2), a molecule for telomere maintenance, the expression of ICAM-1 was increased, whereas endothelial NOS activity was decreased, implying the contribution of senescent ECs to atherogenesis [3]. On the other hand, studies with LDL receptor knockout (KO) atherosclerotic mice showed that the disturbance of flow in the ascending aorta and aortic arch promotes endothelial cell senescence [25]. Moreover, in vitro studies indicated that aberrant flow is a signal that induces cellular senescence [25]. It was also revealed that in EC-specific progeroid mice overexpressing the dominant-negative form of TRF2 under the control of Tie2 or VE-cadherin promoter, SA-β-gal^+^ cells and the mRNA expression of cyclin-dependent kinase inhibitors (p16/INK4, p19/ARF, and p21/WAF-1) were increased in the lung endothelium, and atherosclerosis (lipid accumulation in the aorta and serum) was accelerated with target deletion of the apolipoprotein E (ApoE) gene [26]. Interestingly, a recent study demonstrated that the gene ablation of ECs expressing glycoprotein nonmetastatic melanoma protein B (GPNMB) as a senescent cell antigen reduced the atherosclerotic burden in ApoE KO mice on a high-fat diet [27].

VSMCs, another cellular component of blood vessels, are in a non-proliferative quiescent state in a normal artery; however, in atherosclerotic lesions, the cells become proliferative to prevent the rupture of the fibrous cap. The involvement of senescent VSMCs in the progression stages of atherosclerosis has been suggested since VSMCs are at a low level of proliferation in advanced plaques, even in the presence of molecules that induce the migration and proliferation of VSMCs [4]. SA-β-gal^+^ α-smooth muscle actin (α-SMA)^+^ VSMCs were detected in plaque intima and the advanced fibrous caps of carotid atherectomies, co-immunostained with antibodies for p21/WAF-1 or p16/INK4 [4]. The plaque-associated VSMCs express the features of cell cycle arrest (the decreased expression of cyclin D/E and telomere shortening) and accumulated DNA damage (increased oxidative stress, examined immunohistochemically with anti-α-SMA and anti-8-hydroxy-2’-deoxyguanosine antibodies) [4]. Another report indicated that p53 and p21/WAF-1 were increased in patients’ primary VSMCs isolated from atherosclerotic femoral arteries, compared with cells from healthy arteries [28]. Furthermore, senescent VSMCs expressed IL-6, IL-8, MCP-1, and MMP9 in vitro [29], and SA-β-gal^+^ VSMCs in carotid plaques expressed IL-6, suggesting a causative role of SASP-expressing senescent VSMCs in the progression of atherosclerotic disorders [29]. Moreover, transgenic mice expressing the TRF2 loss-of-function mutant, specifically in VSMCs, showed increased atherosclerosis and necrotic core formation, whereas VSMC-specific functional TRF2 decreased the necrotic core areas [30].

Macrophages and foam cells contribute to the progressive stages of atherosclerosis by secreting proinflammatory cytokines and MMPs. Childs et al. demonstrated that senescent intimal foam cells accumulate in atherosclerotic lesions and function as the key drivers of atheroma formation in LDL receptor KO mice [31]. They showed that on a high-fat diet, plaque-rich aortic arches showed elevated transcript levels of p16/INK4, p19/ARF, and SASP molecules MMPs, IL-1α, and TNF-α. SA-β-gal^+^ cells accumulated in atherosclerotic plaque and demonstrated some ultrastructural features of ECs (elongated cells), VSMCs (spindly cells), and macrophages (large lipid-loaded cells) when analyzed with transmission electron microscopy. Using genetic and pharmacological methods (p16/INK4-3MR and INK-TTAC mice) to eliminate p16/INK4-expressing senescent cells, it was found that senescent foam cells were deleterious at the early atherogenesis stage of plaque initiation and growth, the progression stage of plaque maturation, and the advanced stage of plaque instability [31].

Together, these results suggest that senescent ECs, VSMCs, and foam cells play an atheropromotive role by expressing SASP-related inflammatory molecules. Senescent ECs may promote monocyte recruitment to vascular endothelium via increased MCP-1, ICAM-1, or VCAM-1 in the early stage of atherosclerosis and may increase thrombosis via activated PAI-1. In the progression stage, senescent VSMCs in the plaque intima may accelerate the migration of medial VSMCs via increased IL-6, MCP-1, and MMPs. Finally, senescent macrophages and foam cells may increase plaque vulnerability via increased MMPs, IL-1α, and TNF-α in the progression stage [32].

## 3. Atherosclerosis and LPS

Many studies have proposed a role for bacterial infection in atherosclerosis [33,34,35]. Gram-negative *Chlamydia pneumoniae* (*C. pneumoniae*), *Escherichia coli* (*E. coli*), *Helicobacter pylori* (*H. pylori*), and *Porphyromonas gingivalis* (*P. gingivalis*) are speculated to be involved in disease pathogenesis since these bacteria have often been identified in patients with atherosclerosis via polymerase chain reaction (PCR) analysis or serological tests [36,37]. In addition, epidemiological studies indicate that infection with *C. pneumoniae*, *H. pylori*, or *P. gingivalis* increases the risk of cardiovascular diseases, including atherosclerosis [38,39,40]. Moreover, experimentally, *C. pneumoniae* and *P. gingivalis* infections enhance lesion progression in atherosclerotic mice [41,42].

LPS, an outer membrane component of Gram-negative bacteria, is proposed as a possible pathogenic factor of atherosclerosis that is associated with bacterial infection [43,44]. LPS present in the gut can also translocate into the systemic circulation via gut dysbiosis and changes in gut permeability [45]. In this context, it has been demonstrated that serum LPS levels are increased, and LPS is detected in the atherosclerotic plaques of the carotid arteries of patients. When evaluated with an enzyme-linked immunosorbent assay (ELISA) and immunohistochemistry using anti-LPS antibodies [5,46], the LPS level was significantly higher in serum and positive in the tissue sections of carotid artery plaque from atherosclerotic patients [5]. The presence of LPS was detected as positive in the coronary artery specimens of 11 out of 13 patients with cardiovascular diseases [46]. Moreover, a cohort study (FINRISK 1992) demonstrated that a high serum level of LPS is a risk factor for numerous cardiovascular diseases, including atherosclerosis [47].

To understand the mechanism responsible for the pathogenic effect of bacterial LPS on atherosclerosis, we should note that LPS induces proinflammatory responses in various cell types, including leukocytes and vascular cells. Many in vitro studies have revealed that LPS induces the expression of cytokines, such as TNF-α, IL-1β, IL-6, IL-8, IL-10, IL-12, IL-15, and MCP-1, in monocytes and macrophages [48,49]. Moreover, LPS induces monocyte differentiation and macrophage polarization to the proinflammatory M1 type [50], while LPS induces the Th1-immune responses of T cells that play atherogenic roles [51]. In addition, LPS induces neutrophil activation, accompanied by the downregulation of L-selectin and upregulation of CD1lb/CD18 [52], and suppresses the spontaneous apoptosis of neutrophils, which are recently speculated to be involved in atherosclerosis [53]. In ECs, LPS upregulates the expression of inducible nitric oxide synthase (iNOS), E-selectin, ICAM-1, and VCAM-1 [54]. LPS also induces endothelial actin depolymerization to increase vascular permeability [55] and upregulates the expression of tissue factor [56] which may enhance the prothrombotic state. Moreover, LPS promotes the proliferation of VSMCs and increases the expression of MCP-1, TNF-α, IL-1β, IL-6, and iNOS in VSMCs [57,58,59]. Among these inflammatory molecules induced by LPS, some were found to be increased in LPS-administered atherosclerotic mice. TNF-α, IL-1β, IL-6, and MCP-1 were increased in the plasma and aortic tissue of ApoE KO mice after the injection of LPS for 4 weeks [60,61]. TNF-α, IL-1β, and IL-6 were increased in serum, and VCAM-1 and ICAM-1 were increased in the atherosclerotic plaques of ApoE KO periodontitis mice, accompanied by increased Oil-Red O-stained plaque areas of the aorta after the subgingival injection of LPS for 10 weeks [62]. The underlying signaling pathways important for the LPS-induced inflammatory responses have also been speculated to contribute to pathogenesis in atherosclerosis models [48]. A study using ApoE KO mice demonstrated that the deletion of either toll-like receptor 4 (TLR4) or myeloid differentiation primary response 88 (MyD88), a key cell-surface LPS receptor and mediator of TLR4 signaling, respectively, reduced monocyte adhesion to ECs and eventually prevented plaque formation [63,64]. On the other hand, complete deficiency in TLR2, another receptor for *H. pylori* LPS [31], led to a reduction in atherosclerosis in LDL receptor KO mice [65]. LPS promotes the foam cell formation of macrophages by inducing lipid accumulation and inhibits the phagocytic ability of macrophages by TNF-α production [66,67]. Furthermore, LPS inhibits the expression of Sirt3, which ameliorates lipid deposition and endothelial dysfunction and attenuates atherosclerosis [68,69,70]. In contrast, LPS induces the activation of mTOR, which induces monocyte adhesion and smooth muscle cell proliferation and enhances atherosclerosis [71,72,73].

Based on these observations, it may be reasonable to speculate that in the pathogenesis of atherosclerosis associated with bacterial infection, LPS promotes the early stages of pathogenesis by increasing cytokines/chemokines (TNF-α, IL-1β, IL-6, and MCP-1) and the adhesion molecules (VCAM-1 and ICAM-1) of monocytes and ECs, thereby enhancing the recruitment and adhesion of monocytes to those ECs in which the TLR-mediated LPS recognition and activation of cellular signaling may be involved. Of note, TLR2 and TLR4 expression is increased in human atherosclerotic plaques [74,75] and advanced human atherosclerotic arterial lesions [76], implying that the LPS-induced TLR-mediated inflammatory responses are augmented in the lesion-associated cells of patients.

It remains unclear whether the atherosclerosis-associated bacteria, namely, *C. pneumoniae*, *E. coli*, *H. pylori*, and *P. gingivalis* [33,34], indeed contribute to pathogenesis by sending LPS to host cells as an atheropromotive agent. A constraint to addressing this question may be the technical limitations in detecting bacterial species-specific LPS via immunohistochemistry. Nevertheless, animal experiments suggest that *E. coli* and *P. gingivalis* LPS can influence the pathogenesis of atherosclerosis. Intraperitoneal injection of *E. coli* LPS enhanced the plaque lesion size and macrophage accumulation in ApoE KO atherosclerotic mice [77], and subgingival injection of *P. gingivalis* LPS increased the plaque formation accompanying increased serum inflammatory cytokine levels in ApoE KO mice fed a high-fat diet [62].

To understand the systemic and local mechanisms linking the pathogenic role of LPS in atherosclerosis, it may be important to note that atherosclerosis-associated *C. pneumoniae* [78], *H. pylori* [79], and *P. gingivalis* [80] cause persistent infections in the respiratory tract, gastrointestinal tract, or gingiva, respectively, over many years and have relatively low endotoxic LPS activities [81,82]. This suggests that those bacterial LPS can act on host cells continuously and induce chronic low-grade inflammation. In atherosclerotic patients, these bacterial LPS most probably act on resident cells at the site of infection or circulating blood cells for extended periods, leading to systemic chronic inflammation [35]. It is also possible that these bacteria extravasate from the original foci (respiratory or gastrointestinal tract, or gingiva) and translocate via the circulation to infect the atherosclerotic lesion-associated monocytes/macrophages and vascular cells, where those bacterial LPS may increase local inflammation [35].

## 4. LPS Induces Cellular Senescence

We have described how cellular senescence and bacterial LPS are involved in the pathogenesis of atherosclerosis, yet one critical question remains. How are these two seemingly independent pathways mechanistically linked?

One potential mechanism is the direct induction of cellular senescence by LPS. Previous studies have speculated that LPS has a senescence-inducing effect on various cell types. This theory is based on evidence of the increased expression of cell cycle arrest-associated molecules or inflammatory factors, or the increased SA-β-Gal activity in LPS-exposed cells in vitro (Table 1). For example, LPS (7-day treatment) increased SA-β-Gal activity and lysosomal content and slightly shortened the telomere length in A549 pulmonary alveolar epithelial cells [83]. The LPS of *P. gingivalis* (6-day treatment) increased SA-β-Gal activity, upregulated the expression of p16/INK4, p21/WAF-1, and p53 (molecules for cell cycle arrest), and the proinflammatory molecules ICAM-1, IL-1β, IL-6, IL-8, MCP-1, MMP12, and MMP13 in mouse periodontal alveolar osteocytes [84]. LPS (6-day treatment) induced SA-β-Gal activity and p53 expression and increased the cells in G0/G1 phase (evaluated by the formation of heterochromatic foci) in BV2 mouse microglial cells [85]. The LPS of *E. coli* (6-day treatment) increased SA-β-Gal activity and the expression of p21/WAF-1 and p53 and suppressed cell growth in human dental pulp stem cells [86]. These observations suggest that cells undergo senescence upon exposure to LPS for several days. On the other hand, cellular senescence is induced by LPS, even after 24 h of exposure. LPS exposure for 24 h induced SA-β-Gal activity, gene expression of inflammatory molecules (TNF-α, IL-6, MCP-1, vascular endothelial growth factor-A, and hypoxia-inducible factor-1α) as well as the activation of SASP-related signaling molecules (C/EBPβ, p38 MAPK, and NF-κB p65) in mouse adipocyte progenitor cells [87]. LPS exposure for 24 h increased SA-β-Gal activity and upregulated the expression of molecules for cell cycle arrest (p16/INK4, p21/WAF-1, and p53) and proinflammatory responses (the production of IL-6, TNF-α, and CXCL1) in THP-1 macrophage-like cells [88]. LPS exposure for 24 h also increased p53 and p21/WAF-1 expression in human umbilical vein endothelial cells (HUVECs) [89].

A few reports have suggested the senescence-inducing action of LPS in a non-atherosclerosis mouse model. Chronic LPS inhalation (repeated exposure of *E. coli* LPS aerosol for 10 weeks) of normal and chronic obstructive pulmonary disease mice increased the expression of p21/WAF-1, γ-H2AX, and SA-β-Gal^+^ cells in bronchial epithelial cells [90] or lung tissues [91]. These reports suggest the involvement of LPS in vivo in the cellular senescence of the lung, where pulmonary epithelial cells and macrophages are exposed to low levels of LPS present in the air. The effect of LPS on cellular senescence has not been evaluated in animal models of atherosclerosis. However, the mentioned in vitro studies and accumulation of LPS in atherosclerotic patients speculate that LPS in serum and atherosclerotic lesions induces cellular senescence by directly binding to circulating monocytes and the plaque-associating cells (macrophages/foam cells, ECs, and VSMCs), in which cell surface LPS receptors (TLRs) and the downstream signaling (NF-κB and MAPK pathways) may be involved in SASP expression. Further studies are needed in atherosclerotic mice to confirm the role of LPS in cellular senescence.

## 5. LPS Enhances SASP-Associated Proinflammatory Responses of Senescent Cells

ICAM-1 of ECs plays a pivotal role in the early phase of atherosclerosis [2]. Importantly, the SA-β-Gal^+^-senescent ECs of atherosclerotic plaques highly express ICAM-1 in human carotid arteries [92]. Since both senescent ECs and LPS are detected in atherosclerotic lesions, and there is evidence for a direct effect of LPS on senescence induction, we hypothesized that LPS acts on senescent (SASP-acquired) ECs to enhance their proinflammatory characteristics in atherosclerotic lesions. In this context, LPS may not only induce the senescence of vascular ECs but also augment their SASP proinflammatory response in atherosclerotic lesions. This premise is consistent with the coexistence of LPS and SASP^+^ cells (e.g., high ICAM-1-expressing SA-β-Gal^+^ cells) on the vascular surface of atherosclerotic plaque [92]. Here, we present our results, demonstrating that LPS indeed increases ICAM-1 expression and NF-κB activation in senescent ECs as an SASP-enhancing agent under culture conditions [93].

**Figure 1:**
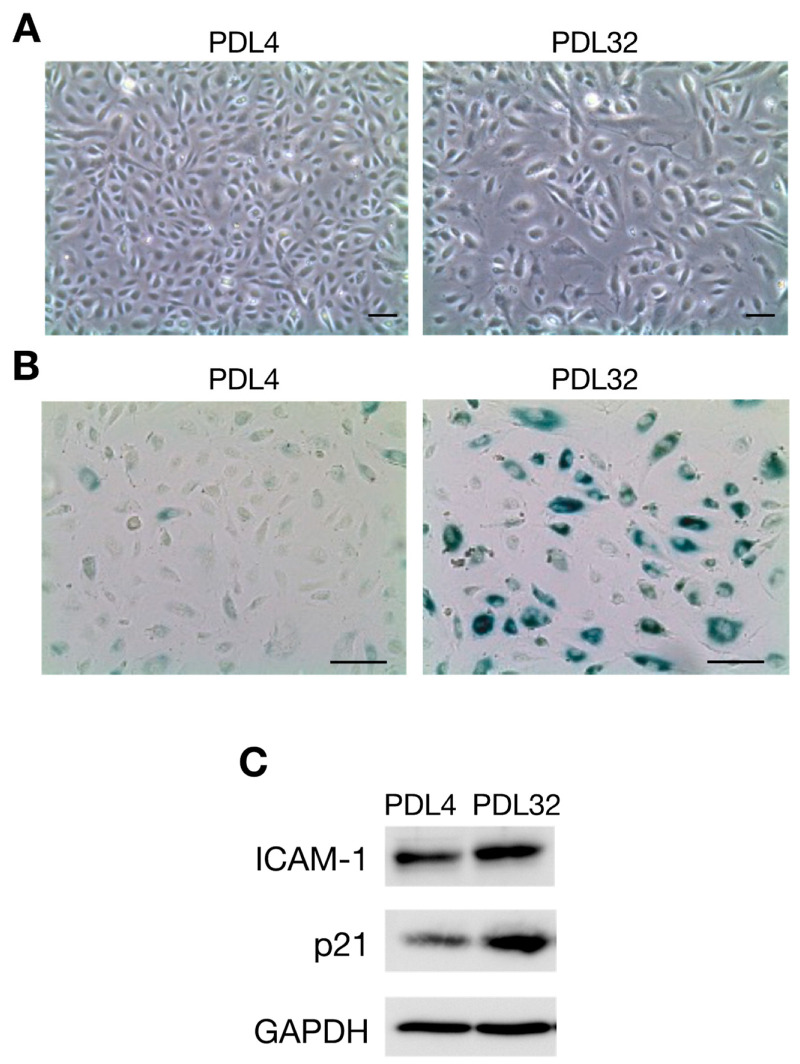
The characteristics of senescent endothelial cells prepared by the serial passage of human umbilical vein endothelial cells (HUVECs). HUVECs were cultured in endothelial cell growth media in 10 cm-diameter dishes and then passaged every 3 or 4 days. (**A**) Phase contrast images of population doubling level 4 (PDL4) (left) and PDL32 (right) cells are shown. PDL is defined as the total number of times that the cells in the population have doubled. PDL32 cells have an enlarged and flattened morphology (original magnification ×50). (**B**) Images showing SA-β-Gal staining with PDL4 (left) and PDL32 (right) cells. SA-β-Gal activity is increased in the PDL32 cells (original magnification ×100). (**C**) The expression of ICAM-1 and p21/WAF-1 in PDL4 and PDL32 cells was evaluated by Western blotting. Images are representative of three independent experiments. Glyceraldehyde 3-phosphate dehydrogenase (GAPDH) expression was detected as an internal control. Scale bars, 100 µm [93].

**Figure 2:**
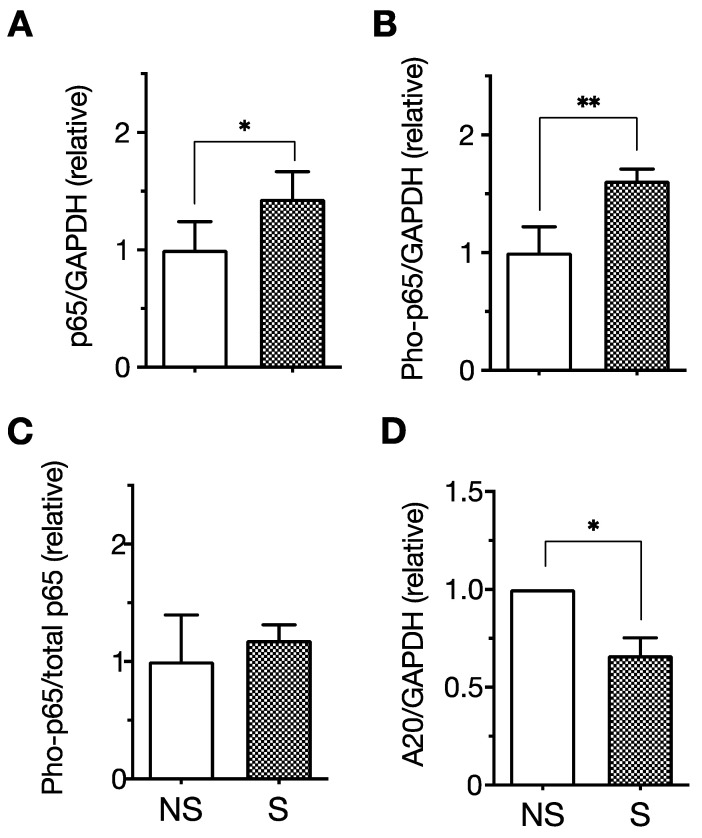
The expression of NF-κB p65 and A20 in senescent endothelial cells. The expression of p65, phosphorylated p65 (Pho-p65), and A20 (a negative regulator of NF-κB) was analyzed with senescent (S) and non-senescent (NS) human umbilical vein endothelial cells (HUVECs) by Western blotting. The relative expression of total p65:GAPDH (**A**), Pho-p65:GAPDH (**B**), Pho-p65:total p65 (**C**), and A20:GAPDH (**D**) of senescent cells was expressed as a ratio to non-senescent cells. Data are the mean ± standard deviation (SD) of six independent experiments. Values were compared between senescent and non-senescent cells. The expression of total p65 and Pho-p65 was upregulated in senescent cells. In contrast, the expression of A20 was downregulated in senescent cells. * *p* < 0.05, ** *p* < 0.01 [93].

We first induced the senescence of ECs by passaging HUVECs repeatedly. As is consistent with previous studies [94,95], the cell proliferation rate gradually decreased with increasing culture period duration until cells no longer proliferated, at approximately 50 days. In association with the end of proliferation, we noted that cell morphology changed from spindle-shaped at population-doubling level (PDL) 4 to enlarged at PDL32 (Figure 1A). An increase in SA-β-Gal activity (Figure 1B) and upregulated p21/WAF-1 expression (Figure 1C), which are representative markers of senescent cells, were also confirmed in PDL32 cells. Moreover, ICAM-1 is recognized as a representative SASP marker of ECs, since the overexpression of ICAM-1 is normally observed in several senescent EC models, including those achieved by stress stimulation [96], serial passage [94], and oncogene transfer [92]. The expression of ICAM-1 (Figure 1C) and the phosphorylation level of the SASP-related signaling molecule NF-κB p65 were increased in senescent PDL32 cells (Figure 2A–C) compared to non-senescent PDL4 cells. In contrast, the expression of A20, a negative regulator of NF-κB [97], was downregulated in senescent cells (Figure 2D).

**Figure 3:**
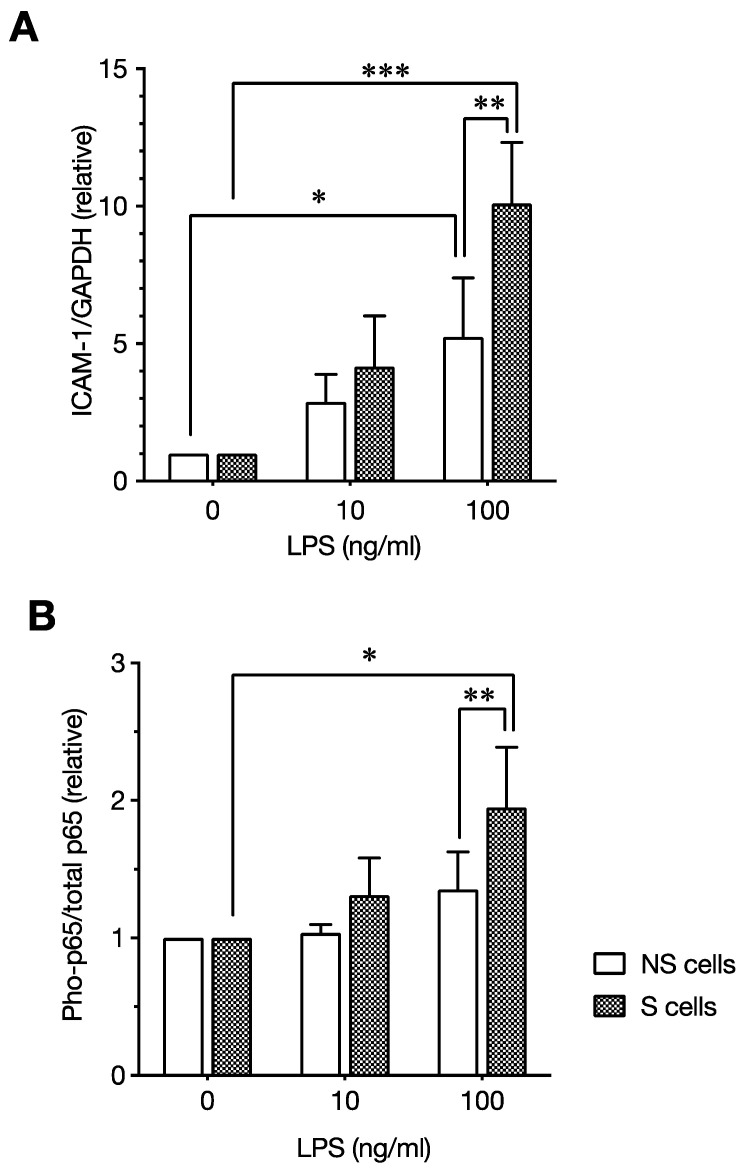
A comparison of the levels of lipopolysaccharide (LPS)-induced ICAM-1 expression and NF-κB p65 phosphorylation in senescent and non-senescent endothelial cells. Senescent and non-senescent human umbilical vein endothelial cells (HUVECs) were incubated with or without LPS (10 or 100 ng/mL) for 24 h, and the expression of ICAM-1, p65, and phosphorylated p65 (Pho-p65) was analyzed by Western blotting. The relative expression of ICAM-1:GAPDH (**A**) and Pho-p65:total p65 (**B**) was expressed as a ratio to control cells (0) incubated without LPS in senescent (S) and non-senescent (NS) cells. Data are the mean ± standard deviation (SD) of four independent experiments. Values were compared with (100 ng/mL) and without (0 ng/mL) LPS incubation, as well as between senescent and non-senescent cells incubated with LPS (100 ng/mL). LPS-induced ICAM-1 expression and Pho-p65 level were enhanced in senescent cells. * *p* < 0.05, ** *p* < 0.01, and *** *p* < 0.001 [93].

**Figure 4:**
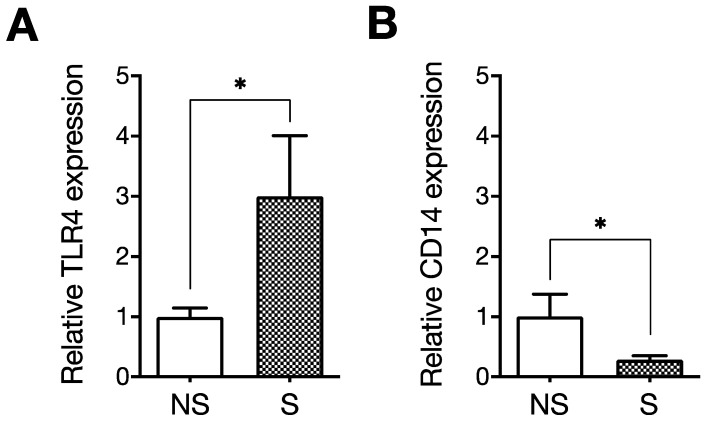
The expression of TLR4 and CD14 in senescent endothelial cells. Flow cytometry was used to analyze the expression of TLR4 and CD14 in senescent (S) and non-senescent (NS) human umbilical vein endothelial cells (HUVECs). The relative expression of TLR4 (**A**) and CD14 (**B**) of senescent cells was expressed as a ratio to non-senescent cells. Data are the mean ± standard deviation (SD) of at least three independent experiments. Values were compared between senescent and non-senescent cells. The expression of TLR4 was upregulated, whereas the expression of CD14 was downregulated in senescent cells. * *p* < 0.05 [93].

Next, we evaluated the effect of LPS on senescent ECs compared with non-senescent ECs. LPS enhanced ICAM-1 expression and NF-κB p65 phosphorylation, not only in the non-senescent cells but also in the senescent cells. Importantly, the effect of LPS was augmented on senescent cells, as ICAM-1 expression and NF-κB p65 phosphorylation were more potently induced in senescent cells compared with non-senescent cells (Figure 3). Moreover, cell surface expression of the LPS receptor TLR4 was significantly increased in senescent ECs (Figure 4A). These results indicate that LPS enhances SASP-associated proinflammatory responses via the NF-κB pathway in senescent ECs, possibly mediated by the increased TLR4. In addition, NF-κB signaling is basically enhanced in the senescent ECs and is likely expanded by LPS. Altogether, these observations suggest that senescent ECs contribute to the pathogenesis of atherosclerosis, via their basal proinflammatory phenotype and enhanced inflammatory responses to LPS.

Recent reports by other groups revealed similar LPS SASP-enhancing action on senescent ECs. They showed that LPS hyperactivates the expression of SASP molecules (IL-6, IL-1β, CCL2, TNF-α, CCL5, CXCL1, and VCAM-1) and signaling molecules (p38 MAPK and p65 NF-κB) in senescent HUVECs that are generated by ionizing radiation [98] and long-term culture [26]. This confirms our speculation that LPS enhances the expression of SASP molecules, including adhesion molecules, cytokines, and chemokines, by enhancing the NF-κB pathway in senescent ECs [93]. In addition, the SASP-enhancing effect of LPS was suggested in other cell types. LPS from *Campylobacter rectus*, associated with adult periodontitis, induced the higher production of IL-6 and plasminogen activator in senescent gingival fibroblasts prepared by serial passage [99,100], while LPS increased the expression of IL-1α, IL-1β, IL-6, MCP-1, and PAI-2 in senescent adipocyte progenitors prepared with ionizing radiation relative to LPS-treated non-senescent cells [101].

Interestingly, it has been reported that TNF-α can induce endothelial senescence [12]. Moreover, angiotensin II and TGF-β induce vascular smooth muscle cell senescence [102,103]. Thus, it may be possible that LPS enhances the SASP-associated responses of senescent endothelial cells and smooth muscle cells.

Although we have not yet acquired in vivo evidence of similar LPS effects in atherosclerotic lesions, our results support the hypothesis that LPS is a crucial factor acting on SASP^+^-senescent ECs, enhancing proinflammatory responses during atherogenesis. In future studies, in vivo LPS binding to senescent ECs and SASP enhancement should be examined in LPS-injected atherosclerotic mouse models.

## 6. Effect of LPS-Neutralizing Peptide LL-37 on Senescent Cells

Given that LPS induces/enhances cellular senescence, it may be reasonable to speculate that LPS-neutralizing agents will inhibit senescent cell formation and activation by LPS. LL-37, of the cathelicidin family, is a human antimicrobial peptide with 37 amino acids and amphipathic α-helical conformation predominantly produced by neutrophils and epithelial cells following cleavage from the precursor of human cationic antibacterial protein of 18 kDa. In addition to the broad spectrum of bactericidal (membrane-disrupting) functions, LL-37 directly binds with LPS, thus neutralizing the biological activity of LPS [104]. We have previously revealed that LL-37 suppresses the LPS-induced IL-1β release and pyroptosis of monocytes/macrophages [105], as well as the LPS-induced apoptosis of ECs [106], by inhibiting LPS binding to the receptors (CD14/TLR4) of these cells. Thus, it is tempting to speculate that LL-37 may inhibit LPS-induced SASP expression in senescent cells. We evaluated the effect of LL-37 on the SASP-enhancing action of LPS in senescent HUVECs that were simultaneously stimulated with LPS and LL-37. In non-senescent ECs, LL-37 almost completely suppressed the LPS-induced expression of ICAM-1 due to its LPS-neutralizing activity. In senescent ECs, LL-37 suppressed the LPS-induced expression of ICAM-1; however, the suppression was partial, and ICAM-1 expression was retained [93] because the expression of inflammatory molecules is basically increased, while the LL-37-unbound LPS likely enhances the inflammatory responses in senescent ECs. Thus, LL-37 could inhibit LPS-induced SASP expression in senescent ECs; still, the inhibitory action is limited.

## 7. Conclusions

Atherosclerosis is classified as a disease of aging since increasing age is an independent risk factor for the development of atherosclerosis, which is promoted by biological aging and cellular senescence [1]. In this review, we presented and discussed a hypothesis related to the senescence-inducing and SASP-enhancing actions of LPS in atherosclerosis. It has been demonstrated recently that the elimination of senescent vascular ECs or foam cells attenuates the atherosclerosis burden in mouse models [27,31]. This confirms an atherogenic role of senescent cells in patients and suggests that controlling bacterial infections and the LPS burden could be a potential approach for decreasing senescent cells and preventing atherosclerosis. To further unveil the role of LPS in the pathogenesis of atherosclerosis as a direct senescence-inducing/enhancing agent, in vivo association of LPS and senescent cells (e.g., the colocalization of labeled LPS and senescent cell markers such as SA-β-Gal or p16/INK4^+^) and the correlations with pathological changes should be analyzed in LPS-injected atherosclerotic mice. In addition, the effect of infection control or LPS removal/neutralization on cellular senescence (particularly of circulating and lesion-associating vascular cells) and the lesion progression should be evaluated in atherosclerotic mice and patients.

## Figures and Tables

**Table 1:** Summary of the senescence-inducing action of LPS.

Bacterial Species of LPS	Condition	Target Cells	Responses				Ref.
			SA-β-Gal Staining	Induction of SASP Factors	Induction of Molecules for Cell Cycle Arrest	Other Responses	
*P. gingivalis*	10 ng/mL, 6 days	Mouse periodontal alveolar osteocytes	+	ICAM-1, IL-1β, IL-6, IL-8, MCP1, MMP12, MMP13	p16, p21, p53	Disordered distribution of F-actin	[84]
*E. coli*	10 ng/mL, 6 days (3 or 6 times)	Human dental pulp stem cells	+	NA	p21, p53	Disordered distribution of F-actin Increased nuclear localization of NF-κB p65	[86]
Species not described	10 ng/mL, 6 days (3 or 6 times)	BV2 mouse microglial cells	+	NA	p53	Increased SAHF formation	[85]
Species not described	15 µg/mL, 7 days	A549 human pulmonary alveolar epithelial cells	+	NA	NA	Increased lysosomal content Telomere shortening	[83]
Species not described	0.2 µg/mL, 24 h	Mouse adipocyte progenitor cells	+	TNF-α, IL-6, MCP1, VEGF-A, HIF-1α	NA	Increased expression of C/EBPβ, p38 MAPK and NF-κB p65	[87]
Species not described	1 µg/mL, 24 h	THP-1 human macrophage-like cells	+	IL-6, TNF-α, CXCL1	p16, p21, p53	Increased expression of NF-κB	[88]
Species not described	1 µg/mL, 24 h	HUVECs	NA	NA	p21, p53	Increased expression of NF-κB p65	[89]

NA: not assessed.
